# Antibiotic resistant Achromobacter xylosoxidans are highly susceptible to bacteriophages and often are killed synergistically by phage/antibiotic combinations

**DOI:** 10.21203/rs.3.rs-9371970/v1

**Published:** 2026-04-30

**Authors:** David Pride, Manola Orlatti, Norhan Turki, Marine Tourneur, Phoolwanti Rani, Jesse Leonard, Natasha Kanhirun, Alisha Blanc, Andrew Su, Yixian Huang, Gabriel Koch, Zachary Pride, Joseph Oh, Monica Bates, Alicia Amamoto, Victoria Carrico, Bethzabe Serrano Buelna, Rob Knight, John Bradley, Douglas Conrad, Ana Cobián Güemes1

**Affiliations:** UCSD; UCSD; UCSD; UCSD; UCSD; University of California San Diego; University of California San Diego; UCSD; UCSD; UCSD; UCSD; UCSD; UCSD; University of California San Diego; UCSD; UCSD; UCSD; UC San Diego; UCSD, Rady Childrens Hospital; UCSD; University of California San Diego

**Keywords:** Bacteriophages, Cystic Fibrosis, antibiotic resistance, Achromobacter, xylosoxidans, MDR, XDR, extensively drug resistant

## Abstract

Achromobacter infections pose significant challenges to people with Cystic Fibrosis (CF). This microbe often becomes multidrug resistant (MDR) or extensively drug resistant (XDR), leaving physicians with few antibiotic treatment options. It is a growing problem in people with CF because it is difficult to eliminate with antibiotics, leading to chronic lung infections that increasingly acquire antibiotic resistance. We have been searching for alternative therapies for Achromobacter infection and identified a cadre of 26 bacteriophages (viruses that attack bacteria) that target it that could allow us to reduce reliance on antibiotics. We tested these phages against a collection of 56 MDR and XDR clinical Achromobacter isolates and found that all were susceptible to multiple of these phages. We also evaluated whether we could restore the activity of certain antibiotics by using them as adjunctive therapy to phage treatment in vitro. We found that when meropenem (a beta lactam antibiotic) was added to phage treatment, susceptibility to meropenem was demonstrated in many isolates regardless of whether the Achromobacter was initially susceptible to meropenem. The data presented here suggests that combination therapy using meropenem with an active phage should be considered for treatment of Achromobacter infections regardless of pre-existing antibiotic susceptibility.

## IMPORTANCE

Achromobacter infections are a rising threat to those with Cystic Fibrosis and other chronic pulmonary disease due to their ability to acquire resistance following exposure to antibiotics. Achromobacter accumulates resistance to antibiotics because resistant strains of Achromobacter persist in the same individual over time while becoming increasingly multi-drug resistant (MDR) or extensively drug resistant (XDR) following exposure to new antibiotics, driving the need to develop alternative therapies for subsequent treatment. We identified a large group of bacteriophages that kill Achromobacter and found that they killed all clinical isolates within our MDR and XDR collection. Moreover, we also tested whether the combination of antibiotics and phages could result in the reversion of meropenem MICs from “resistant” to “susceptible” regardless of whether preexisting meropenem resistance existed. Antibiotic-phage combinations resulted in conversion to meropenem susceptibility based on assessment of MICs in many instances as long as phage susceptibility was present. This indicates that prior meropenem susceptibilitywas not a requirement to achieve antibiotic-phage synergy. Thus, phage treatments for Achromobacter infections can potentially be instituted in combination with antibiotics such as meropenem regardless of antibiotic susceptibility testing results.

## INTRODUCTION

Antibiotic resistant bacteria present a serious and growing challenge as their numbers continue to increase across the globe^1^. Nonfermenting gram negative bacilli are among the most antibiotic-resistant^2^. The constant evolutionary battle between competing bacteria has promoted the emergence of several mechanisms of antibiotic resistance^3^, including enzymatic destruction of antibiotics and mutations in the intracellular targets within bacteria; changes in cell wall structure to prevent antibiotic entry, and efflux pumps that allow the organisms to move potentially lethal compounds out of the cytoplasm. *Achromobacter* spp. contain virtually all these mechanisms of antibiotic resistance^4^.

Patients with chronic infections, particularly those with Cystic Fibrosis (CF), provide anatomic locations within the lower respiratory tract in which bacteria like Achromobactercan exist and evade host defenses and antibiotic entry^5^; the bronchiectatic lung in CF may contain large pockets of infected mucus within injured lung. Mucus plugs may also create conditions within airways and lungs that allow bacteria to survive^6^, providing the opportunity for repeated invasion of adjacent airways and lung^7,8^. Repeated antibiotic treatment for recurrent infection allows expression and persistence of resistance mechanisms noted above^9^, creating antibiotic-resistant pathogens that force us to direct our attention to other means of killing bacteria, including the use of Achromobacter bacteriophages^10^. In addition, Achromobacter is an emerging pathogen in bloodstream infections^11^.

Phages have been used as experimental treatments for antibiotic resistant bacterial infections, usually when they are applied alongside required standard-of-care (SOC) antibiotics, even if the bacterial pathogen is resistant to all SOC antibiotics^12^. In these situations, it often is not known whether there are cooperative effects (additive or synergistic) between the antibiotics and the phages or whether there may be antagonism. There also is very little guidance to suggest which antibiotics and phages to use in combination to treat infections in such patient populations^13^. In this work we tested the effects of phages and antibiotic combinations in *Achromobacter* spp. infections using several antibiotics with different mechanisms of action. We discovered that certain phages combined with specific antibiotics were likely to result in synergistic interactions against the *Achromobacter* spp. even when there was no evidence of prior antibiotic susceptibility.

## RESULTS

### Achromobacter collection.

In an effort to identify a diverse collection of Achromobacter strains, we collected a group of isolates from UCSD hospitals between 2022 and 2025. These isolates mostly represented *Achromobacter xylosoxidans* and had varying antimicrobial susceptibilities. We collected 56 isolates in total, with 23 qualifying as multi-drug resistant (MDR) and 33 qualifying as extensively drug resistant (XDR). Many of these were isolated from people with CF, however, a number were isolated from individuals with other acute and chronic conditions (**Table S1**). Many of these Achromobacter infections repeatedly failed antibiotic treatment and were often recurrent in nature, demonstrating the need to develop alternative treatment strategies aside from antibiotics alone.

### Isolation and characterization of phages.

**Figure 1:**
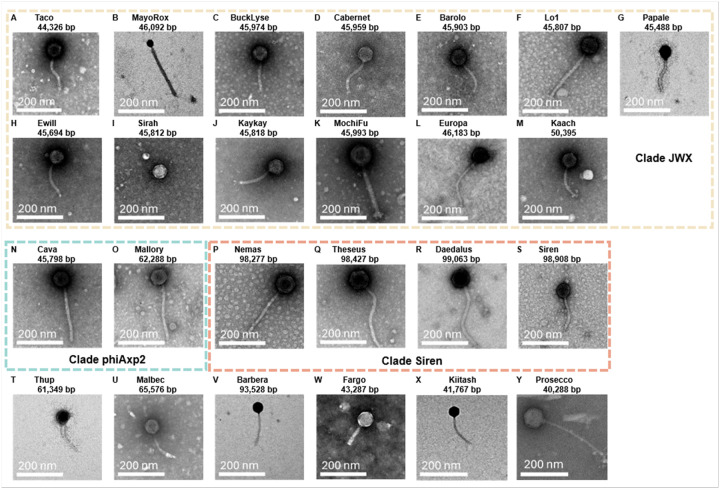
Achromobacter Phages Morphology. Transmission Electron Microscopy of 25 phages. Phages are organized by their phylogenetic clades, Clade JWX (A-M), Clade phiAxp2 (N, O), Clade Siren (P-S), monophyletic phages (T-Y).

While there are bacteriophages capable of killing *Achromobacter* spp., we did not have access to any of those phages. We therefore engaged in a phage hunting expedition to identify phages with strictly lytic potential against Achromobacter isolates (**Table 1**). We identified 26 unique phages against Achromobacter that demonstrated relatively diverse morphologies. Via electron microscopy, we imaged each of the phages and found morphologies consistent with myoviruses, siphoviruses, and podoviruses (Figure 1).

**Figure 2:**
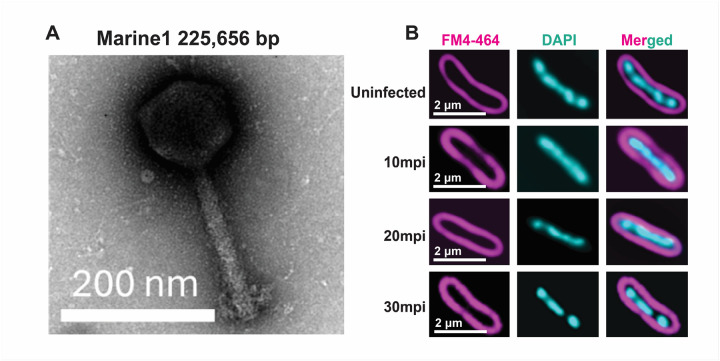
Achromobacter jumbo phage Marine1 morphology and infection phenotype. A) Transmission Electron Microscopy of jumbo phage Marine1. B) Fluorescence microscopy of *Achromobacter xylosoxidans*AX49 cells, either uninfected or infected with the jumbo phage Marine1. Images were acquired at the indicated time points post-infection. Cell membranes were stained with FM4–64 (magenta) and nucleic acids with DAPI (cyan). Time is denoted as minutes post-infection (mpi).

We sequenced the genomes of each of these phages to verify that they were unique and to classify them according to their genetic sequences. We confirmed their genome sizes ranging from 40.3kb to 225.7kb (**Table 1**). The largest of the phages, Marine1, represented a jumbo phage (Figure 2-A), but did not have a nucleus-like structure as determined by DAPI staining (Figure 2-B). We classified each of the phages via phylogenetic analysis and found that many were similar yet distinct from previously identified Achromobacter phages (**Figure 3**). They spanned nearly every clade of previously identified Achromobacter phages available. Four phages belonged to a previously undescribed clade that we called Siren. An integrase was identified in phage Thup, but no other integrases or other genes suggesting lysogeny were identified in the other phages. The jumbo phage Marine1 did not form a cluster with a prior Achromobacter jumbo phage Motura. When placed into a phylogenetic structure including only other previously identified jumbo phages (**Table S2**), it clustered most closely with a Ralstonia jumbo phage RSL2 (**Figure S1**). Both Achromobacter and Ralstonia are Betaproteobacteria.

### Host ranges.

**Figure 4:**
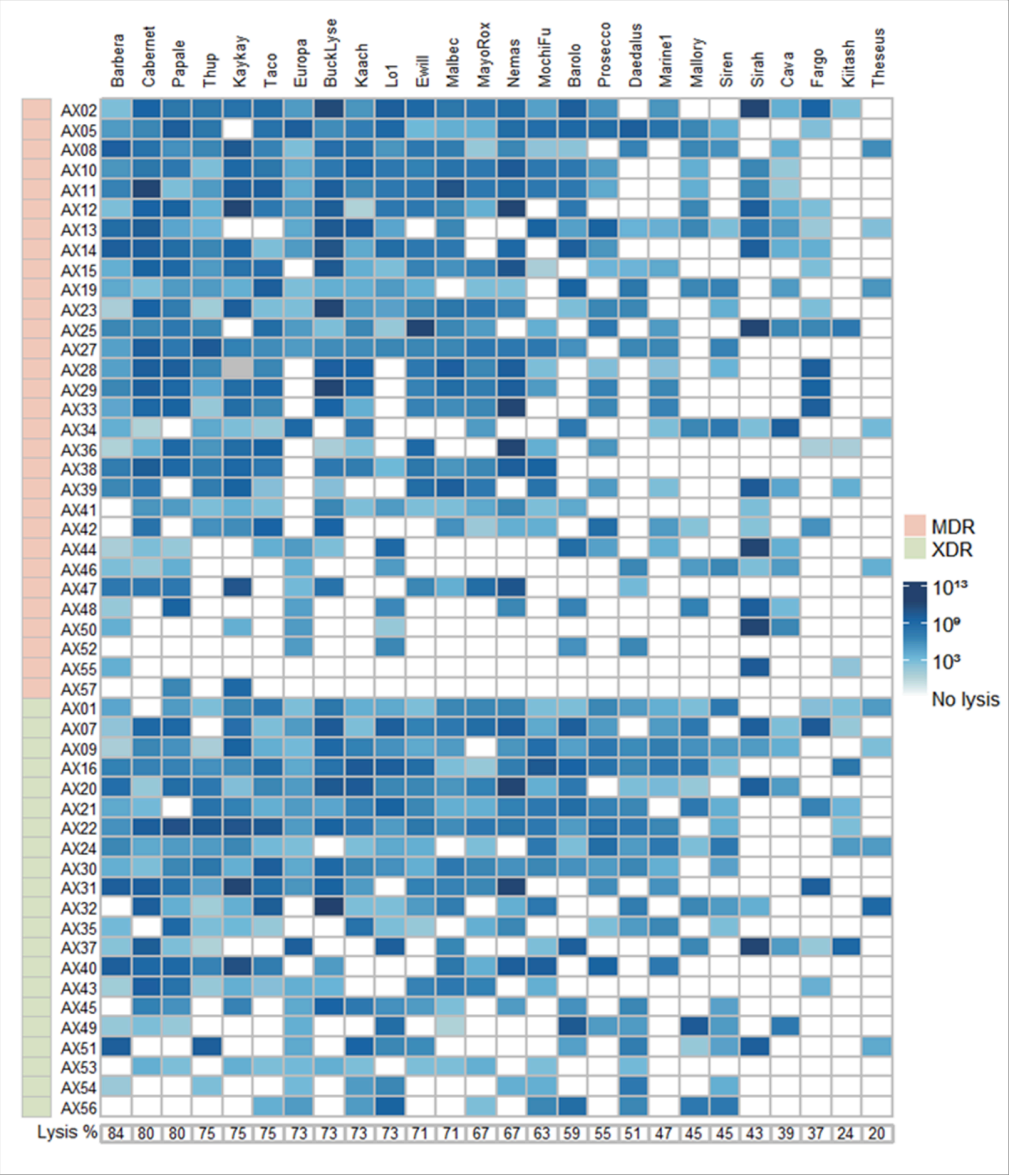
Host Range of 26 Achromobacter phages isolated in this study vs 50 clinical strains of MDR or XDR *Achromobacter xylosoxidans* and the reference strain AX19. Phage infection was tested in 0.3% LB agar plates using 12 phage dilutions.

Given the breadth of this phage collection, we evaluated whether they were capable of efficiently killing our Achromobacter isolates. We used quantitative plaque assays to assess the susceptibility of each isolate to each phage. We found that for all of the isolates, there was at least one phage capable of lysing it efficiently (Figure 4). Phage Barbera showed the broadest host range, killing 84% of the tested bacteria strains. For most isolates, there were multiple phages capable of killing them, and all of the phage lysis could be diluted to single plaques and recovered, which excluded the potential that there was lysis from without. Interestingly, all of the MDR and XDR isolates could be killed efficiently by phages, suggesting that antibiotic resistance and phage susceptibility were uncoupled.

### Phage-Antibiotic synergy.

Because Achromobacter infections persist in the CF lung despite proper antibiotic therapy^6^, we tested whether there might be cooperativity between antibiotics and phages to assist with reducing organism burden beyond that achievable with antibiotics alone. We set up grided arrays in 96-well plates where each well had the same number of bacteria, but the amount of phage or antibiotic added differed in concentration gradients across the y-axis and x-axis, respectively (**Figure 5 A-C**). We performed these assays for multiple Achromobacter isolates using multiple different phages and antibiotics.

We focused primarily on the antibiotic meropenem because it represents a common treatment for Achromobacter infections although many of our MDR and XDR isolates demonstrated resistance to it (**Table S1**). Our primary goal was to determine whether we could identify cooperativity between the phages and the antibiotic in a manner that resulted in reduced MICs to meropenem, indicating synergy between the phage and the antibiotic. We found evidence of synergistic interactions for *A. xylosoxidans* strain AX31 with phages Bucklyse and Kaach (**Figure 5, Panels A, B, D, E, G, and H**). We did not find evidence of synergy when phage Taco was used with meropenem (**Panels C, F, and I**). We also did not identify evidence of synergistic interactions with strain AX19, largely due to resistance to phage Bucklyse (**Figure S2, Panels A, D, and G**), or due to exquisite susceptibility to phages Kaach (**Figure S2 Panels B, E, and H**) and Taco (**Figure S2 Panels C, F, and I**).

**Figure 6:**
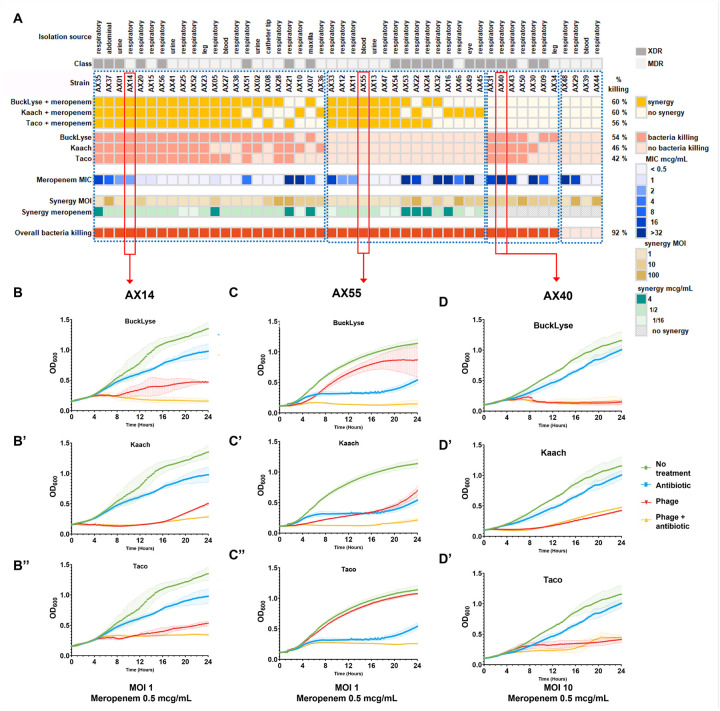
Summary of Phage-Antibiotics-Synergy between meropenem and phages BuckLyse, Kaach or Taco in 50 clinical strains. A) bacteria killing by phages and antibiotics (yellow scale) or phages alone (orange scale) in liquid over 24 hours; meropenem MIC for each clinical isolate determined by the clinical laboratory (blue scale); MOI (brown scale) and meropenem concentration (green scale) at which synergy was observed. Overall bacteria killing (red scale). B) Representative growth curves of synergy in which phages plus antibiotics show greater bacteria killing than each one separately. C) Representative growth curves of synergy in which the phages show moderate to no bacteria killing by themselves, but there is strong bacteria killing with the addition of antibiotics. D) Representative growth curves of phages capable of bacteria killing, but there is no synergy with the addition of antibiotics.

We also tested most of the *A. xylosoxidans* isolates for evidence of synergistic interactions between phages and meropenem (Figure 6, **Panel A**) and found that 77% of them demonstrated evidence of synergy (Figure 6, **Panels B, B’, B”, C, C’, C”**). Of those that did not demonstrate evidence of synergy, 14% were exquisitely susceptible to the phages making it difficult to demonstrate synergy (Figure 6, **Panels D, D’, D”**), and 8% were resistant to the phages tested and meropenem. The synergistic interactions between phages and meropenem were interesting because when the combination of antibiotics and phages were used, the MIC for the combination was significantly reduced below the CLSI recommended breakpoint for meropenem (4 mcg/mL). That we identified the phenomenon for multiple Achromobacter isolates, and for Achromobacter phages Bucklyse, Kaach, and Taco, indicates that these results were not specific to an individual phage in our collection.

Our initial synergy screening included 4 antibiotics: two beta lactam antibiotics that inhibit cell wall synthesis (meropenem and ceftazidime), one antibiotic that inhibits folate production therefore blocking DNA and proteins synthesis (trimethoprim-sulfamethoxazole), and one fluoroquinolone which inhibits DNA replication (levofloxacin). In addition to identifying synergy with meropenem, we also identified synergistic interactions with ceftazidime (**Figures S3 and S4**). Some synergistic interactions were observed between phage Kaach and trimethoprim-sulfamethoxazole, but not with other phages (**Figures S5 and S6**). We did not identify synergistic interactions between the three phages tested and levofloxacin (**Figures S7 and S8**). While these results indicate that synergistic interactions across different antibiotic mechanisms are possible with phages, they were not consistent with any of the antibiotics tested aside from beta lactams.

## DISCUSSION

There are a number of pathogens that are prone to acquiring antibiotic resistance and become significant problems in immunocompromised, long-term care, and chronically ill populations such as people with CF. Even with the use of CFTR modulators there is still chronic bacteria colonization in CF patients^14^. *A. xylosoxidans* has become a significant problem due to its ability to become both MDR and XDR, and particularly in the CF population where it becomes a chronic colonizer, this feature of the organism can result in deadly outcomes^5,6,8^. Because *A. xylosoxidans* accumulates resistance as the human host is exposed to antibiotics over time, it appears inevitable that it will become MDR and/or XDR^7,15^. Phage therapy has the potential to addresses the unmet need for alternative Achromobacter anti-infective therapy^12,16,17^.

To address the *A. xylosoxidans* problem at our institution, we developed a collection of phages that specifically target the organism. The collection of 26 phages showed complete coverage of all the tested patient-derived MDR and XDR *A. xylosoxidans* in our collection. This finding suggested that nature already has provided a solution to the complex problem of MDR and XDR *A. xylosoxidans* infections; we just haven’t been developing that solution. Just finding phages capable of killing these antibiotic-resistant microbes wasn’t necessarily the end game. Antibiotics remain the standard of care for bacterial infections and won’t be easily replaced by phages. Thus, we evaluated whether there may be complementary interactions between antibiotics and phages.

We have identified synergistic interactions between antibiotics and phages before^18,19^, as have others^20,21^, so we anticipated that we may find some complementary interactions between antibiotics and phages in the killing of *A. xylosoxidans*. We focused on meropenem (Figure 6) because it is an often-used anti-Achromobacter drug for serious infections, but we observed somewhat similar results for other antibiotics as well (**Figures S3-S6**). Synergy between meropenem and phages has been reported in *Klebsiella pneumoniae*^22^, *Acinetobacter baumanii*^23^, and *Pseudomonas* aeruginosa^24^.

We observed significant inhibition of the bacteria when antibiotics and phages were used in combination. In many cases, the bacteria were resistant to the antibiotics, but susceptibility was restored when used in combination with phages. This is similar to what we observed in the *Enterococcus* spp.isolates for several different antibiotics^19^. These observations suggest that it is unnecessary to test antibiotic susceptibilities for *A. xylosoxidans* when using phage treatments in every situation because the use of the phages in combination with meropenem could restore antibiotic susceptibility. While these principles have not been demonstrated in patients, they are highly reproducible *in vitro* and will be the subject of future clinical trials to identify the effects of combination antibiotic-phage treatments on bacterial clearance for hosts such as *A. xylosoxidans*.

## METHODS

### Achromobacter antibiograms.

Strains used in the study were isolated from patients at the UCSD Center for Advanced Laboratory Medicine. The antibiotic susceptibility testing was performed on the isolates using MicroScan NM46 and DNM2 panels (**Table S1**). MDR or XDR classifications were defined as previously described^25^.

### Phages isolation.

Fifty milliliters of fresh wastewater was centrifuged at 5,000 rpm for 20 minutes and the supernatant was collected. The supernatant was syringe filtered using a 0.2 μm filter. One milliliter of filtered wastewater was incubated at 37 °C for 16 hours with 20 μL of 0.2 OD600 bacteria (*A. xylosoxidans* AX19) in 2 mL of LB media supplemented with 10 mM MgCl_2_ and 10 mM of CaCl_2_. After co-incubation, the liquid was 0.2 μm filtered, and eight serial dilutions were made. Two microliters of each serial dilution were spotted on a bacteria lawn of 0.3% LB agar supplemented with 10 mM MgCl_2_and 10 mM CaCl_2_. The plates were incubated for 16 hours at 37 °C. Individual plaques were picked and plaque purified 3 times. After plaque purification, a phage stock was produced in liquid culture.

### Jumbo phage isolation.

Wastewater was centrifuged at 3,000 × g for 10 minutes and the supernatant was collected, then centrifuged at 4,696× g for 55 minutes. The supernatant was discarded and the pellet was resuspended in 10 mL of SM buffer and gently vortexed. An overnight culture of *Achromobacter xylosoxidans* AX49 was diluted to 0.2 OD600, 200 μL of culture was combined with 4 mL of warmed 0.3% LB top agar and poured over 1% LB plates. The plates were dried for 15 minutes near an open flame. Ten microliters of processed wastewater were spotted and dried under an open flame for 30 minutes. Plates were incubated upside down overnight at 37°C. Phages formed ~1 mm plaques on LB top agar (0.3%) over the bacterial lawn. Phage plaques were picked using a pipette tip and gently stabbed repeatedly into an LB plate with 1% agar. This area was then streaked with a loop, changing loops with each streak. Molten 0.3% LB top agar and 100 μL of overnight 0.2 OD600 AX49 culture were combined and poured onto the plate starting from the lowest dilution point to the stabbed area. The top agar was then allowed to sit at room temperature for 15 minutes before being incubated overnight at 37°C. Plaques were purified 3 times.

### Phage morphological characterization.

Phage lysates were concentrated using a 10 KDa MWCO cellulose filter (Amicon, Millipore Cat# UFC9010). Concentrated phage lysates (titer > 10^9^ PFU/mL) were used for imaging. A carbon-coated grid (PELCO SynapTek Grids, product# 01754-F) was placed on a drop of 40 μL of phage lysate. Grids were washed with DI water twice and then negatively stained with 2% uranyl acetate (pH 4.0) for 45 seconds. Imaging was performed using JEOL 1400 plus located at the University of California, San Diego—Cellular and Molecular Medicine Electron Microscopy Core (RRID: SCR_022039).

### Fluorescence microscopy for phage infection.

Fluorescence microscopy was performed in at least two independent biological replicates. For infection, 100 μL of overnight-grown host cells (*A. xylosoxidans* AX48) was infected with 100 μL of jumbo phage Marine1 and incubated for 10 minutes at 37°C. The fixative was prepared by combining 1 mL of 16% paraformaldehyde with 1 μL of 25% glutaraldehyde and mixing thoroughly. Forty microliters of this fixative were added to 200 μL of exponentially growing cells, along with 8 μL of 1× PBS, followed by gentle mixing. Cells were incubated at room temperature for 20 minutes.

Following fixation, cells were pelleted at 6,000 × g for 2 minutes and washed three times with 1× PBS. The final pellet was resuspended in 20 μL of staining solution prepared in 25% LB, supplemented with DAPI (from a 2.5 mg/mL stock; Thermo Fisher Scientific, Cat# D1306) and FM4–64 (from a 3.75 μg/mL stock; Thermo Fisher Scientific, Cat# T13320). Cells were stained for 5 minutes at room temperature. Stained cells were then mounted on 1% agarose pads prepared in 25% LB on single concave slides and immediately imaged.

Live-cell imaging was performed using a DeltaVision Elite Deconvolution microscope (Applied Precision, Issaquah, WA, USA). Immediately before imaging, a glass coverslip was placed on top of the agarose pad. For static fluorescence microscopy, imaging pads were stained with 8 μL of dye mixture (25 μg/mL DAPI and 3.75 μg/mL FM4–64) at room temperature for 1 minute prior to imaging (DAPI: Thermo Fisher Scientific, Cat# D1306; FM4–64: Thermo Fisher Scientific, Cat# T13320). Images were deconvolved using DeltaVision SoftWoRx software (version 6.5.2).

### Phage genome characterization.

Two hundred microliters of phage lysate were treated with 3 μL of DNaseI (2000 u/ml) and 30 μL of DNAse buffer and incubated at 37 °C for 30 minutes, followed by 10 minutes at 70 °C. DNA extraction was performed using Monarch Mag Viral DNA/RNA extraction kit (NEB, #T4010S), the final elution was performed in 100 μl of nuclease free water. DNA was quantified with Qubit HR DNA (cat #Q33231, Invitrogen). Fifty microliters of fragmented DNA were used for sonication using Covaris (microTUBEs, duty cycle 10%, intensity 5, cycles per burst 200, time 180 seconds). Sequencing libraries were prepared using NEBNext Uktra II DNA library prep kit for Illumina (NEB #E7645S) using 50 μL of fragmented DNA as input and 10 amplification cycles. Libraries were sequenced in an Illumina iSeq instrument as PE150. Reads were *de novo* assembled using SPADEs through the webserver BV-BCR^26^. Assembly graphs were inspected and, in most cases, a single contig was present. When multiple contigs were present, the closest reference genome was used as scaffold and manually assembled using MAUVE^27^ visualization. Phage CDS were identified using PHANNOTATE^28^ and annotated through the webserver BV-BCR^26^. Average Nucleotide Identity (ANI)^29^ was calculated between the sequenced phages, if two phages share more than 99% ANI, they were considered the same phage and the phage stock with the higher genome coverage was kept for further analysis. Phage genome annotations were screened for integrases, excisionases and toxin genes (**Table 1**).

### Phage Proteomic Tree.

Newly isolated Achromobacter phage genomes were compared to publicly available Achromobacter phages (n=47, **Table S1**) through a Phage Proteomic Tree approach using VIPtree^30^.

### Host range evaluation.

Phage host range was evaluated by spot titer. A bacteria lawn was established using 450 μL of 0.2 OD600 bacteria mixed with 6.75 mL of molten 0.3% LB top agar and poured onto 1.5% LB plates (150 mm). Two microliters of each serial dilution were spotted and dried in a hood. Plates were incubated for 24 hours at 37 °C. After incubation, phage titer and spot turbidity were recorded.

### Phage-antibiotic synergy in liquid media.

A 1:1000 subculture was started from an overnight culture of bacteria in LB media. OD600 was monitored until 0.1 was reached. An antibiotic was added to a 96-well plate in 30 μL of sterile water, to achieve concentrations ranging from 64 mcg/mL to 0.016 mcg/mL, and no antibiotic. Phages were added in 30 μL of LB media to achieve MOIs of 0, 0.1, 1, 10 or 100. One hundred and forty microliters of bacteria were added to each well in the plate. Each condition was tested in triplicate. Negative controls as well as no treatment controls were included in triplicate. The plates were incubated for 24 hours at 37 °C with shaking in a LogPhase600 reader, and OD600 was measured every 15 minutes with growth curves generated in GraphPad Prism (version 10.6.1). OD600 at 24 hours was used to calculate the percentage growth for each condition relative to the no treatment controls. The OD600 of each replicate of the treatment condition was divided by the average of the no treatment wells and multiplied by 100. The average of the triplicates was used to obtain the mean relative percentage growth for each treatment. These values were displayed as heatmaps using R (version 4.5.2). Pairwise differences in percentage growth between treatment groups were assessed using an independent two-sample t-test, which was implemented using the compare_means function implemented in the R package ggpubr (version 0.6.0). Statistical significance was displayed in the figures. Significance thresholds were determined as follows: p ≤ 0.05 (*), p ≤ 0.01 (**), p ≤ 0.001 (***), while p > 0.05 not significant (ns).

All strains were evaluated for phage-antibiotics-synergy between meropenem and phages BuckLyse, Kaach or Taco independently using the liquid media method described above. Three antibiotic concentrations were used: 4 μg/mL, 0.5 mcg/mL, and 1/16 μg/mL. MOIs of 1,10, or 100 were used. Synergy was determined by using the end point OD600 measurements as percentage inhibition in the highest single agent model.

## Supplementary Material

This is a list of supplementary files associated with this preprint. Click to download.

- AchromobacterTable13102026DP.docx
- AllSupplementalFiles.pdf

Tables are available in the Supplementary Files section.

## Data Availability

All phage genomes have been deposited in NCBI Genbank (**Table 1**).

## Abbreviations

CF: cystic fibrosis
MDR: multi-drug resistant
XDR: extensively drug resistant
MIC: minimum inhibitory concentration
LB: Luria Beltran broth
TEM: transmission electron microscopy
MOI: multiplicity of infection
